# Highlights on the Application of Genomics and Bioinformatics in the Fight Against Infectious Diseases: Challenges and Opportunities in Africa

**DOI:** 10.3389/fgene.2018.00575

**Published:** 2018-11-27

**Authors:** Saikou Y. Bah, Collins Misita Morang’a, Jonas A. Kengne-Ouafo, Lucas Amenga–Etego, Gordon A. Awandare

**Affiliations:** ^1^West African Centre for Cell Biology of Infectious Pathogens, University of Ghana, Accra, Ghana; ^2^Vaccine and Immunity Theme, MRC Unit The Gambia at London School of Hygiene & Tropical Medicine, Banjul, Gambia

**Keywords:** bioinformatics, genomics, infectious diseases, antimicrobial resistant, diagnosis

## Abstract

Genomics and bioinformatics are increasingly contributing to our understanding of infectious diseases caused by bacterial pathogens such as *Mycobacterium tuberculosis* and parasites such as *Plasmodium falciparum*. This ranges from investigations of disease outbreaks and pathogenesis, host and pathogen genomic variation, and host immune evasion mechanisms to identification of potential diagnostic markers and vaccine targets. High throughput genomics data generated from pathogens and animal models can be combined with host genomics and patients’ health records to give advice on treatment options as well as potential drug and vaccine interactions. However, despite accounting for the highest burden of infectious diseases, Africa has the lowest research output on infectious disease genomics. Here we review the contributions of genomics and bioinformatics to the management of infectious diseases of serious public health concern in Africa including tuberculosis (TB), dengue fever, malaria and filariasis. Furthermore, we discuss how genomics and bioinformatics can be applied to identify drug and vaccine targets. We conclude by identifying challenges to genomics research in Africa and highlighting how these can be overcome where possible.

## Introduction: Omics and Bioinformatics in Infectious Diseases

**FIGURE 1:**
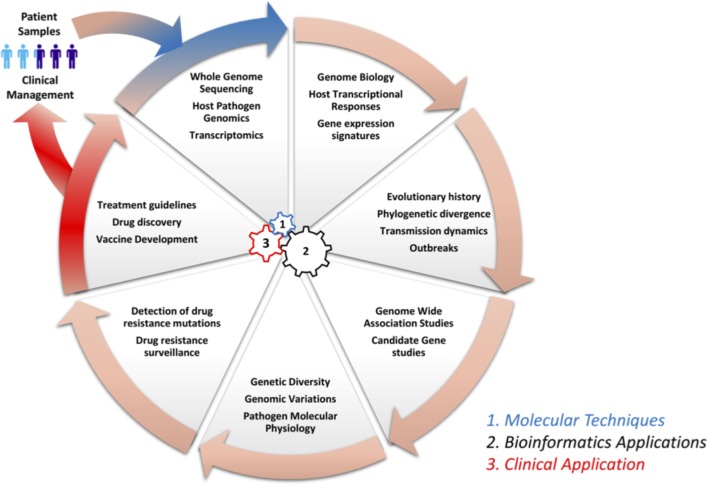
Circle of infection disease genomics/bioinformatics. This shows a representation of genomic laboratory technologies, bioinformatics analysis, and potential applications. This includes: (1) molecular techniques such as whole genome sequencing by methods like Illumina to generate sequence reads, which are needed for the (2) bioinformatics application to study host systemic responses, pathogen genomics, and transmission dynamics. Further, bioinformatics can be applied to determine genetic diversity, investigation of drug resistance mechanisms and surveillance, and the identification of vaccine targets in systems vaccinology. Finally (3), all this information can be integrated to define treatment guidelines and patient management.

Genomics and bioinformatics have contributed immensely to our understanding of infectious diseases: from disease pathogenesis, mechanisms and the spread of antimicrobial resistance, to host immune responses. Herein, we review some of the major contributions of genomics and bioinformatics in infectious disease research using examples of three diseases that account for large proportions of morbidity and mortality as well as a neglected tropical disease. Specifically, we review *M. tuberculosis*, which causes TB, a disease responsible for approximately two million deaths globally per year. Dengue virus (DENV) causes Dengue fever, which is a re-emerging mosquito borne viral disease, responsible for more than 350 million cases annually (WHO, 2017; World Health Organization Western Pacific Region, 2018). *Plasmodium falciparum* causes malaria, a parasitic disease that accounts for the highest morbidity and mortality in Sub-Saharan Africa, especially in children under five and pregnant women (WHO, 2018b), and Filariasis, which is a neglected tropical disease. Figure 1 shows a circular wheel of genomics/bioinformatics as can be applied in infectious diseases as discussed herein, ranging from understanding host and pathogen genome biology to genome-wide association studies (GWAS) as well as the identification of drug targets and drug resistance surveillance to patient management. This encompasses molecular techniques, bioinformatics and clinical applications (Figure 1). We also highlight the application of genomics and bioinformatics to the identification of vaccine targets and drug discovery. We conclude by highlighting some challenges of conducting bioinformatics research in resource-limited countries in sub-Saharan Africa.

## Omics of Tuberculosis Pathogens and Host Responses

Tuberculosis caused by members of the *M. tuberculosis* complex is a leading cause of death, with about 9 million cases and two million deaths per year globally (WHO, 2018a). The mycobacterial genome was first sequenced in 1998 and many more *M. tuberculosis* genomes have since been sequenced (Cole et al., 1998; Guerra-Assunção et al., 2015; Yun et al., 2016). These genomes provide great avenues for the genomic characterization, development of improved diagnostic tools, drug susceptibility testing, and molecular epidemiology of circulating mycobacterial strains. Host-pathogen genomics and transcriptomics have over the past decade enhanced our understanding of human-mycobacterium interactions and in the identification of potential diagnostic and prognostic markers (Anderson et al., 2014; Maertzdorf et al., 2015).

An understanding of the *M. tuberculosis* genome biology is invaluable in the control of TB. The *M. tuberculosis* genome is GC rich and consists of about 4000 genes and, unlike other bacteria, a large proportion of its genome encodes proteins and enzymes involved in lipogenesis and lipolysis (Cole et al., 1998), reflecting its thick lipid cell wall. TB control is hampered by antimycobacterial resistance, multidrug resistance (MDR) and, recently, extensively drug resistant (XDR) mycobacterial strains (Leisching et al., 2016). Genomics analysis has immensely contributed to the identification of drug resistance-conferring mutations and surveillance (Köser et al., 2013). Whole genome analyses have demonstrated that mycobacterial drug resistance is largely attributed to single nucleotide polymorphisms (SNPs); for example, rifampicin (RIF) resistance arises from mutations in the *rpoB* gene and mutations in the *katG* and *inhA* lead to isoniazid resistance (da Silva et al., 2011). Newly characterized genetic mutations in *M. tuberculosis* genomes have also been shown to play key roles in the emergence of antimycobacterial drug resistance (Sun et al., 2012). Analyses of 161 drug resistant *M. tuberculosis* genomes identified 72 genes, 28 intergenic regions and 21 SNPs with strong and consistent associations with drug resistance (Zhang et al., 2013). Genomic analysis has also identified lineage mutation rate differences and predicted the emergence of antimycobacterial resistance (Ford et al., 2013). A retrospective analysis of thousands of *M. tuberculosis* genomes collected from African and European patients identified 120 resistance-determining mutations for first and second line antimycobacterial drugs, which could be valuable in developing new assays for drug susceptibility testing (Walker et al., 2015). Furthermore, genomics through the use of GWAS has been used to identify novel mutations associated with resistance to cycloserine, ethionamide, and para-aminosalicylic acid, suggesting the involvement of efflux pump in the emergence of resistance (Coll et al., 2018). A number of genomics-based tools have been developed to detect drug resistance including Mykrobe Predictor, PhyResSE, and TB-Profiler, which are easy to use by researchers with no bioinformatics expertise and can predict drug resistance within minutes after obtaining sequences (Bradley et al., 2015; Coll et al., 2015; Feuerriegel et al., 2015). Mykrobe Predictor has a sensitivity and specificity of 82.6 and 98.5%, respectively (Bradley et al., 2015). TB-Profiler was developed using a mutation library consisting of 1,325 mutations in different genes associated with drug resistance in 15 anti-tuberculosis drugs and had more than 75% sensitivity as well as more than 90% specificity for all drugs tested (Coll et al., 2015). A recent study evaluating the performance of these tools showed that their sensitivity ranges from 74 to 80% along with a specificity of more than 95% (van Beek et al., 2018). However, there is still a need for optimization of analysis pipelines to make them applicable in field settings where the disease burden is usually the highest.

Genomics analysis has also been used to determine the evolutionary history and spread of mycobacterial strains such as the Beijing strain, demonstrating its spread from the Far East (Merker et al., 2015). An investigation of *M. tuberculosis* transmission dynamics is important in monitoring outbreak; Mehaffy et al. (2014) demonstrated that whole genome analysis can be used to monitor infections to decipher transmission dynamics. Furthermore, genomics has also been applied to decipher transmission dynamics of *M. tuberculosis* in Vietnam, suggesting that SNPs in ESX-5 type VII secreted protein EsxW could potentially contribute to enhancing transmission (Holt et al., 2018). Furthermore, genomics has been applied to investigate TB outbreaks, genotyping of the outbreak associated lineages, and their evolution during the outbreak (Jamieson et al., 2014; Stucki et al., 2015). Indeed, analysis tools have been developed for the prediction of *M. tuberculosis* spoligotypes from raw sequence reads, and in combination with other analysis tools also determine antibiotic resistance as well as transmission dynamics (Coll et al., 2012; Bradley et al., 2015). Some genomics methods can also be employed to identify mixed infections as well as infections with a single strain and have recently been applied to clinical isolates from Malawi (Sobkowiak et al., 2018).

Genome-wide association study (GWAS) has also been used to identify candidate gene variants associated with susceptibility to active tuberculosis. GWAS analyses in African patients from Ghana, Gambia, Uganda and Tanzania identified TB disease-associated SNPs located on three chromosomal loci: 18q11, 11p13, and 5q33 (Thye et al., 2010, 2012; Sobota et al., 2016). Similarly, GWAS studies have also been done in Europe identifying SNPs in the *ASAP1* gene on chromosome 8q24 and in a genomic region in which class II human leucocyte antigen (HLA II) is encoded (Curtis et al., 2015; Sveinbjornsson et al., 2016). Recently, a GWAS study in a Han Chinese population also found SNPs in mitofusin-2 (*MFN2*), regulator of G protein signaling 12 (*RGS12*) and HLA II beta chain to be associated with active TB (Qi et al., 2017). This highlights that host genetics play significant roles in susceptibility to active TB and may explain why some individuals remain latently infected while some develop active TB despite having similar exposure levels. Furthermore, based on host genetic variants, GWAS analysis could be applied to identify latently infected individuals who are at a high risk of developing active TB for preventative interventions. Once validated, identified SNPs can be used to develop point of care diagnostics to identify high risk people for mass preventative treatment.

Host transcriptomics are increasingly being used to understand systemic responses to infections and to identify diagnostic and prognostic markers. Mistry et al. (2007) were among the first to use microarray technology to study host systemic response to TB, identifying a nine gene-signature with potential for TB diagnosis. Jacobsen et al. (2007) applied microarray analysis to investigate the host pathway biology and potential diagnostic biomarkers. Analyzing peripheral blood mononuclear cells (PBMCs), they found a monocyte-derived gene expression signature identifying CD64, lactoferrin and Ras-Associated GTPase-33A as potential diagnostic biomarkers, which were further validated in another independent study population in South Africa (Maertzdorf et al., 2011). Applying gene set enrichment analysis to microarray gene expression identified metabolic pathways such as insulin metabolism, immune cell differentiation and inflammation in TB (Lesho et al., 2011). A neutrophil-driven interferon signature consisting both type I and type II interferon during TB was also identified using microarray analysis (Berry et al., 2010). The type I interferon pathway was also observed by Ottenhoff et al. (2012) identifying IL15RA, UBE2L6, and GBP4 as the main molecules involved. A 393-transcript signature for active TB and an 86-transcript signature with a potential for distinguishing TB from other inflammatory diseases were also identified (Berry et al., 2010). In addition, a biosignature consisting of 27 transcript signatures to distinguish active from latent TB and 44 transcript signatures to distinguish active TB from other diseases were recently identified (Kaforou et al., 2013). Microarrays have also been used to demonstrate that host transcriptional responses to *M. africanum* and *M. tuberculosis* differ following treatment (Tientcheu et al., 2015), which could be important in the management of patients infected with the different mycobacterial strains. Furthermore, host gene expression has also been used to monitor treatment responses and predict treatment outcome, which will be valuable in testing new drug regimens and new antimycobacterial drugs (Thompson et al., 2017). These studies prove the potential of host genomics in providing a better understanding of disease pathophysiology, prognosis and host pathway biology in response to an infectious agent.

In addition, arrays have also been applied to childhood TB, to identify signatures for active tuberculosis and a signature that distinguishes active tuberculosis from other diseases in sub-Saharan Africa (Anderson et al., 2014). Similarly, a 9-gene signature was also identified in Warao Amerindian children, further highlighting the potential of using host biomarkers for TB diagnosis (Verhagen et al., 2013). Host transcriptional analysis is moving from array-based technologies to RNA sequencing and has been applied to 16 gene signatures that identified people with a high risk of developing TB 2 years before diagnosis in sub-Saharan Africa (Zak et al., 2016). However, it is noteworthy that identified biosignatures have a variable number of genes, from about 10 to more than 100, and there is very little overlap between some signatures. It will be valuable to conduct a meta-analysis of available datasets to increase statistical power and identify high confidence signatures across studies regardless of circulating pathogens and local environmental factors. In doing such analysis, confounders due to technologies, age and circulating endemic pathogens can be accounted for to give a strong as well as diagnostic and prognostic signature. These studies highlight the potential application of genomics and bioinformatics to interrogate host response for the diagnosis and prognosis of TB, which will contribute immensely to curbing TB morbidity and mortality.

## Dengue Virus Research in the Era of Bioinformatics

Dengue virus (DENV) is a pathogenic single-stranded RNA virus that belongs to the flavivirus genus, which comprises other known pathogenic viruses such as West Nile, yellow fever, Japanese encephalitis, St. Louis encephalitis, tick-borne encephalitis, Omsk hemorrhagic fever and Zika virus (Gould and Solomon, 2008). The re-emergence, evolution, diversity and geographic distribution of flaviviruses make them interesting pathogens (Moureau et al., 2015). Phylogenetic analysis of divergence times suggests that flaviviruses originated from a common ancestor (100,000 years ago) and later split into mosquito and tick borne flaviviruses about 40,000 years ago (Holbrook, 2017). Approximately 40% of the world population is at risk of DENV infection with more than 350 million cases reported annually.

Illumina SNPs genotyping and SNPs identified through whole genome analysis have been used in case-control GWAS statistical analysis to identify SNPs that predispose or confer protection against DENV infection (de Carvalho et al., 2017). The DENV shock syndrome (DSS) has been shown in a GWAS analysis of SNPs in a cohort of 2008 pediatric cases to have a strong association (*P* < 0.5 × 10^-8^) with the human major histocompatibility complex (*MHC*) (rs3132468) on chromosome 6 and phospholipase C (rs3740360 and rs3765524) on chromosome 10 (Khor et al., 2011). Dang et al. replicated the study in 917 Thai children with DSS and confirmed that alleles rs3132468 [MHC I chain related protein A (*MICB*)] and rs3765524 [phospholipase C epsilon 1 (*PLCEI*)] predispose Southeast Asians to DSS (Dang et al., 2014). In contrast, Whitehorn et al. (2013) genotyped 3,961 confirmed cases and 5,968 controls and found that rs3132468 *MICB* and rs3740360 alleles *PLCEI* were associated with less severe phenotypes of DENV infection in both infants and adults. This implies that the effect of these SNPs could be population-specific. Other candidate genes include dendritic cell-specific intracellular adhesion molecule (*ICAM*)-*3* grabbing non-integrin (*DC-SIGN*), C-Type Lectin Domain Containing 5A (*CLEC5A*), immunoglobulin gamma constant fragment receptor (*FCGRIIA*), Toll-Like receptors (*TLRs*), Tumor necrosis Factor (*TNF*), Interferons (*IFNs*), 2′-5′-oligoadenylate synthase (*OASs*), Janus Kinase (*JAK*), Stimulator of Interferon Genes (*STING*), cytokines, chemokines, *ICAM-1* and tryptase 1 proteases (de Carvalho et al., 2017).

Whole genome sequencing (WGS) and phylogenetic methods have been used to investigate DENV outbreaks. Faria et al. (2017) analyzing 92 viral genomes from DENV patients during the 2012 outbreak in Rio de Janeiro, found that at least two thirds of infections went unnoticed and their analysis highlighted the scale of the epidemic spread of DENV after the outbreak. Ahn et al. (2015) investigated the genetic variations in 8,826 nucleotide sequences of whole-genome DENV virus, and demonstrated that there was a distinctive genetic pattern between the four DENV subtypes across different regions (American, Oceanian, Asian, and Africa).

Analyses of envelope encoding nucleotide sequences from India have shown a shift from DENV subtype III to subtype IV, suggesting some level of positive selection (Manakkadan et al., 2013). These phylodynamic methods, which indicate evolutionary process or patterns of genetic diversity of the DENV virus, have also been reconciled with the virus epidemiology so as to decrease the variation between the two methods that are mainly used to study the population dynamics or viral behaviors (Pybus et al., 2012; Rasmussen et al., 2014). Due to the importance of genomics and bioinformatics in viral research, a range of tools has been developed to analyze viral genomes and make inferences (Stamatakis, 2014; Brody et al., 2017).

The use of RNA folding, structural predictions and functional studies has shown that genetic variation of the DENV occurs in nature due to high rates of recombination and error-prone RNA polymerases. A deleterious DENV genome was first shown by Aaskov et al. (2006) whereby a stop codon in the envelope coding region resulted in a defective DENV. Li et al. (2011) also discovered defective interfering viral particles by analyzing short fragments of DENV, suggesting that they may be part of a broader disease attenuating process mediated by the deleterious virus and the defective interfering particles are important in viral replication, thereby enhancing the overall transmission capability of DENV (Li and Aaskov, 2014). Structural RNA predictions have implicated other elements in modulating replication of the virus, such as the downstream cyclization sequence (Friebe et al., 2012), cis-acting elements occurring in the capsid coding region (de Borba et al., 2015), and elements in the promoter Stem Loop A (SLA) and non-structural protein 5 (NS5) regions (Gebhard et al., 2011).

Understanding intra- and inter-host genetic diversity was previously mired with experimental and analytical methods that did not fully account for errors in viral amplifications. Thai et al. (2012) used various statistical approaches to correct for the artefactual mutations resulting from PCR amplifications and sanger sequencing, and showed that the genetic diversity index (Pi) of the DENV was low, ranging from 0 to 0.0013. This suggested sequence conservation, but they were able to show mixed infections and phylogenetically distinct DENV lineages present within the same host. Furthermore, genome-wide scans for patterns of intra-host diversity in DENV identified variants between genes suggesting significant differences in intra-host diversity of the virus in the Nicaraguan population (Parameswaran et al., 2012). Functional annotation of the variants showed the impact of viral mutations on protein function, which strongly suggested purifying selection across transmission events.

Deep sequencing, RNA structural analysis and fitness evaluation have been used to determine processes that DENV employs for host specialization (mosquito or human) using RNA elements in the 3′-UTR (Villordo et al., 2015). A host adaptable stem loop structure was found to be duplicated, which DENV uses to accumulate mutations that are beneficial in one host and deleterious in another host, but the duplication confers a robust mechanism during host switching (Villordo et al., 2015). Recently, Waman et al. (2016) used population genetics methods to compute the genotype diversity and evolution of 990 DENV genomes, and revealed that the DENV-2 population is subdivided into 15 lineages. Their study also indicated the presence of intra-genotype diversity and that the population structure of DENV-2 is spatiotemporal, shaped by episodic positive selection and viral recombination (Waman et al., 2016). The application of genomics and bioinformatics in the study of DENV shows the complexity of the virus biology, which can be exploited in target identification for drug discovery and vaccine development (Guy et al., 2016; Low et al., 2017).

## Progress in Malaria Genomics

Malaria incidence and mortality rates decreased by 21 and 29%, respectively, between 2010 and 2015 (WHO, 2018b). The genetic landscape of *P. falciparum*, the main cause of malaria, is increasingly being unraveled by using deep sequencing to identify polymorphisms and structural and copy number variations, which are fundamental for parasite evolution (Kwiatkowski, 2015). Sequencing consortia such as the MalariaGEN improve our understanding of genomics of both the Anopheles vector and the plasmodium species^1^. A recent study on genotyping accuracy using deep sequencing of *Plasmodium* parental generations and their progenies revealed that polymorphism frequencies can be used as markers of high recombination rates (Miles et al., 2016), which is an important contributor to enhancing immune evasion and drug resistance. Using whole genome deep sequencing and micro-array analysis, a study observed 18 deletions on regions encoding multigene families that are associated with immune evasion (Bopp et al., 2013). The authors showed the presence of chromosomal crossovers in six of the deletions and were able to estimate mutation rates of *P. falciparum* (Bopp et al., 2013).

Bioinformatics has contributed to our understanding of resistant mechanisms to previous drugs such as chloroquine and the emerging resistance to artemisinin-based combination therapies (ACT). Robinson et al. deployed next generation sequencing to investigate multi-clonality, population genetics and drug-resistant genotypes (Robinson et al., 2011). More recently, WGS was used to discover that mutations in the Kelch propeller domain (K-13) are associated with ACT resistance in Cambodia (Ariey et al., 2014; Straimer et al., 2015). Profiling of the drug resistance genes [*P. falciparum* chloroquine resistance transporter (*pfcrt)*, *P. falciparum* multidrug resistance (*pfmdr1*), *P. falciparum* dihydrofolate reductase (*dhfr*) and *P. falciparum* dihydropteroate synthetase (*dhps*), and *P. falciparum* Kelch protein 13 (*pfk13*)] was done using Illumina next generation sequencing and demonstrated that the resistance-associated K-13 variants were largely absent in Africa (MalariaGEN Plasmodium falciparum Community Project, 2016; Nag et al., 2017).

Furthermore, bioinformatics tools have been used to demonstrate multi-locus linkage disequilibrium and local diversity, recent selection through integrated haplotype scores, regional gene flow and allele frequency differentiations (Duffy et al., 2017). Intra-host diversity can now be statistically characterized using the F*ws* metrics because sequencing platforms are able to generate read count data. Auburn et al. characterized within host diversity in 64 samples from West Africa, capturing a multiplicity of infections, number of clone ratios, clonal variation and within-host diversity (Auburn et al., 2012). Bioinformatics analysis of deep sequencing revealed large-scale genetic variations in *P. falciparum* (86158 SNPs), and genome wide allelic frequencies, population structure, linkage disequilibrium and intra-host diversity (Manske et al., 2012). The genetic diversity of *P. falciparum* is dependent on directional and balancing selection, whereby drug pressure and host immunity are the major selective agents, respectively (Mobegi et al., 2014; Duffy et al., 2015).

Genomics has been used to discover novel malaria resistance loci in humans, which provide 33% protection from severe malaria (Malaria Genomic Epidemiology Network, 2015). In Ghana, GWAS identified two unknown genetic loci associated with severe malaria: 1q32 within the ATPase Plasma Membrane Ca^2+^ Transporting 4 (*ATP2B4*) gene and the 16q22.2 linked to a tight junction protein known as *MARVELD3* (Timmann et al., 2012). Most recently, GWAS was used in a longitudinal surveillance to detect K-13 signatures, which led to the identification of a Kelch variant that is suggested to be a potential modulator of artemisinin resistance (Cerqueira et al., 2017).

The *Plasmodium* pathophysiology is increasingly being explored using transcriptomics and proteomics. Bioinformatics and statistical models have been used to describe the genome-wide translational dynamics of *P. falciparum*, showing that parasite transcription and translation are tightly coupled presenting a broad and high resolution of parasite gene expression profiles (Caro et al., 2014). ChIP-Seq and RNA sequencing have been used for polysome profiling to understand the regulation of *Plasmodium* gene expression in humans. Bunnik et al. (2013) observed a delay in peak polysomal transcript abundance for several genes as compared to the mRNA fraction, which they reported to be alternative polysomal mRNA splicing events of non-coding transcripts.

DNA microarray technologies had been used to describe the gene expression patterns of *P. falciparum* during the intra-erythrocytic stage (Bozdech et al., 2003), gametocyte (Young et al., 2005), sporozoite (Siau et al., 2008), liver stage (Tarun et al., 2008), and even between three different strains (Llinás et al., 2006). Recently, microarrays have been used to characterize parasite transcriptomes during cerebral and asymptomatic malaria, which revealed some differentially expressed genes encoding proteins involved in protein trafficking, Maurer’s cleft proteins, transcriptional factor proteins and several hypothetical proteins (Almelli et al., 2014). RNA sequencing has also been used to describe *P. falciparum* expression profiles at different time points and has found novel gene transcripts, alternative splicing events and predicted untranslated regions of some genes providing further information on the parasite biology (Otto et al., 2010). Yamagishi et al. (2014) simultaneously analyzed the human host and the parasite transcriptomes using RNA sequencing, and showed that several human and parasite genes such as Toll-like receptor 2 and TIR domain-containing adapter molecule 2 (*TICAM2*) correlated with clinical symptoms. RNA sequencing has also been employed to study the transcriptome of *P. vivax*, which revealed a hotspot of *vir* genes on chromosome 2, new gene transcripts and the presence of species-specific genes (Zhu et al., 2016). It would be valuable to compare this data with similar data from other related *Plasmodium* species to identify species-specific transcriptomes. Analyzing the transcriptome of Chloroquine sensitive and resistant parasites identified 89 upregulated genes and 227 downregulated genes that were associated with resistance (Antony et al., 2016). These differentially expressed genes are involved in immune evasion mechanisms, pathogenesis, and various host-parasite interactions and could be targeted for drug and vaccine development.

Currently, single-cell RNA sequencing is revolutionizing the study of cell-to-cell heterogeneity. For example, the use of this method led to the discovery of novel variations in the expression of specific gene families that are involved in host-parasite interactions among asexual populations (Reid et al., 2018). Altogether, these studies demonstrate the profound impact of malaria parasite transcriptomics and genomics on our understanding of the parasite (Lee et al., 2017), and identify possible candidate targets for drugs, vaccines and diagnostics (Ludin et al., 2012; Hoo et al., 2016).

## Genomics Research in Filariasis

Filariasis is a neglected chronic disease caused by tissue-dwelling nematodes (filariae) with onchocerciasis and lymphatic filariasis (LF), causing significant health concerns with a disease burden approaching 86 million cumulatively (WHO/Department of Control of Neglected Tropical Diseases, 2016). Onchocerciasis is caused by *Onchocerca volvulus* while LF is caused by three different parasites, namely *Wuchereria bancrofti*, *Brugia malayi*, and *Brugia timori* (Taylor et al., 2010). Elimination of filariasis is challenging because of the unavailability of sensitive diagnostic tools, lack of appropriate treatments and inadequate control measures in resource limited countries.

The *W. bancrofti* and *O. volvulus* genomes have been sequenced, providing opportunities for further genomic analyses (Desjardins et al., 2013; Cotton et al., 2016). Bioinformatics revealed the presence of gene coding for host immune system regulators such as human-like autoantigens as well as serine and cysteine protease inhibitors (Molehin et al., 2012; Cotton et al., 2016).

Molecular studies coupled with computational analyses have demonstrated an association between human host factors and filariasis clinical manifestations. LF infections have been shown to cluster in some families using pedigree studies (Cuenco et al., 2004; Chesnais et al., 2016). These studies show that genetic factors are involved in the regulation of LF infections and affect both the presence and intensity of microfilariae. However, a GWAS would be more comprehensive to demonstrate this genetic susceptibility to LF as has been the case for a tropical lymphedema (Podoconiosis) of non-filarial origin (Tekola Ayele et al., 2012). It is worth mentioning that lymphedema, or elephantiasis, is one of the main features of LF and normally occurs as a result of a compromised lymphatic system (Addiss, 2010). As opposed to LF, which is infectious, Podoconiosis is a non-communicable disease caused by soil particles such as aluminum and silica predominant in volcanic regions (Price, 1976; Davey et al., 2007). A comparative genomics-based study of LF would help to better understand these clinical manifestations.

Most of the pathological features of LF are associated with human-immunogenetics (Taylor, 2003; Junpee et al., 2010), which has been investigated using genomics and bioinformatics. Gene candidate-based genomics studies carried out in Thailand revealed that polymorphisms in the *TLR-2* gene (-196 to -173 deletion, +597 T > C and +1350 T > C) have a strong linkage disequilibrium and were associated with increased risk of asymptomatic LF (Junpee et al., 2010). In a functional study, individuals with the -196 to -173 deletion were found to have significantly low transcription levels compared to those with the wild-type gene (Junpee et al., 2010). Further analyses showed strong association of a mutation (M196A) in human tumor necrosis factors (TNF) receptor-II with hydrocele development, while the A288S mutation of endothelin-1 (ET-1) correlated with low ET-1 1 plasma levels and elephantiasis (Panda et al., 2011).

Population genetics is very important for assessing and understanding the epidemiology and transmission dynamics of filarial diseases (Small et al., 2016; Doyle et al., 2017). Population genomics of *O. volvulus* samples collected from different geographical zones – West Africa (WA), Uganda and Ecuador – demonstrated some level of population structure between WA and other populations (Choi et al., 2016). Furthermore, phylogenetic signals indicative of gene flow and genetic admixture between WA forest and savanna populations were identified. These signals could serve as markers to delineate forest from savanna populations and/or sort out admixed populations (Choi et al., 2016). A study using both nuclear and mitochondrial sequences identified regions in the *W. bancrofti* genome that exhibited an arrangement which was consistent with both balancing and directional selection (Small et al., 2016).

The control of filariasis in general is difficult due to the complex parasite life cycle. In an attempt to demystify the complex life cycle of the parasite, RNA sequencing has been used to investigate gene expression profiles of different developmental stages of *Brugia malayi* (Choi et al., 2011). Transcriptomics analyses revealed stage-specific gene expression correlating with stage-specific pathway activation. Upregulated proteins included cathepsin L and Z-like cysteine proteases that were previously demonstrated to be essential for larva molting in *O. volvulus* (Lustigman et al., 2004) and cuticle and eggshell remodeling in filarial nematodes in general (Guiliano et al., 2004). Another study using a filarial microarray chip composed of 18,104 gene probes revealed that gene expression in *B. malayi* infective larvae (L3s) depends on environmental factors (Li et al., 2009). The gene expression patterns in irradiated L3s, laboratory-adapted L3s and those collected from mosquitoes were found to be different. Gene Ontology analyses showed that upregulated genes in laboratory-adapted and mosquito-derived L3s were mostly involved in growth and invasion, whereas those in irradiated L3s were enriched with immunogenic proteins and proteins involved in radiation repair (Li et al., 2009). Such high throughput genomics analysis is important for understanding the biology/development, invasion, and immune evasion mechanisms of the parasite and could help improve disease control measures (Choi et al., 2011).

Mass drug treatment with Ivermectin (IVM) or Mectizan^®^ and Albendazole is the main strategy for filariasis control in Africa and has been going on for decades (Amazigo, 2008). However, cases of drug resistance have been reported and genomic methods are increasingly being used to investigate mechanisms of resistance. Genotyping and sequencing studies have shown an association between SNPs in some *O. volvulus* genes (P-glycoprotein-like protein, β-tubulin) and the development of resistance (Nana-Djeunga et al., 2012; Osei-Atweneboana et al., 2012). P-glycoprotein was recently demonstrated to be associated with resistance to IVM in a horse filarial species (cyathostomins) with transcript levels measured by RNA-Seq and confirmed by RT q-PCR found to be significantly higher in the resistant compared to sensitive worm population (Peachey et al., 2017). Moreover, GWAS demonstrated that reduced sensitivity of *O. volvulus* to IVM is accounted for by genetic drift and soft selective sweeps. Pooled next generation sequencing of *O. volvulus* worms collected from Ghana and Cameroon repeatedly treated with IVM and phenotypically characterized into poor responder (PR) and good responder (GR) parasites identified genetic variants that considerably delineate GR and PR parasites. One of these variants (SNP, OM1b_7179218) was common in both Cameroon and Ghana worm populations, whereas the others were country-specific (Nana-Djeunga et al., 2014; Doyle et al., 2017). These variants were found to be grouped in quantitative trait loci (QTLs) in which published genes associated with IVM resistance were scarcely found. Gene Ontology^2^ analysis revealed that genes found in those QTLs regions were linked to pathways involved in neurotransmission, development, and stress responses (Harris et al., 2004; Doyle et al., 2017). The involvement of neurotransmission is a promising finding here because one of the main targets of IVM is a ligand-gated channel at neuromuscular junctions (Cully et al., 1994).

The molecular mechanism of Ivermectin is not clearly understood and has been investigated using bioinformatics approaches. RNA-Seq analyses of ivermectin-challenged *B. malayi* adult female worms revealed that genes involved in cell division (meiosis) and oxidative phosphorylation were drastically downregulated as early as 24 h post-exposure (Ballesteros et al., 2016). A similar study in which the worms were instead challenged with flubendazole (FLBZ), a potential macrofilaricide, demonstrated the effect of FLBZ on embryogenesis and cuticle integrity (O’Neill et al., 2016a). Expression of cuticle-related genes and those involved in mitosis or meiosis were notably affected by the treatment. These studies further elucidate the drug-induced inhibition of embryogenesis and microfilarial release from the female worm uterus during larval development as previously demonstrated (O’Neill et al., 2015, 2016b). Knowledge of this mechanism could help in drug repurposing whereby drugs known to have a similar mode of action or mechanism, but are used for the treatment of other parasitic diseases, could be tested for their efficacy on filarial parasites.

## Application of Omics to Vaccine Target Identification and Drug Discovery

The availability of whole genome sequences of both the host and pathogens in different databases such as GenBank^3^ (Benson et al., 2004), EuPathDB (^4^formerly ApiDB), WormBase^5^, Virus Pathogen Database and Analysis Resource (ViPR) has led to tremendous advances in the search for new drug and vaccine targets (Yan et al., 2015; Xia, 2017). This enables high throughput *in silico* screening for the identification of vaccine and drug targets, thus focusing expensive laboratory screening on selected high affinity targets. Though not yet fully implemented in Africa, omics technologies and bioinformatics analyses have aided significantly in the generation of new knowledge toward drug and vaccine target discovery (Yan et al., 2015; Xia, 2017). Genomic, transcriptomic and proteomic analyses of pathogens such as *filariasis* parasites have identified new potential biomarkers that can be invaluable in diagnostics, vaccine and drug development (Armstrong et al., 2016; Bennuru et al., 2017). Kumar et al. (2007), using genome wide *C. elegans* RNA-interference data as proxy, identified a set of 3,059 essential genes in the *B. malayi* genome, from which 589 were characterized as potential drug targets. The prioritization algorithm helps in the prediction of the efficacy, selectivity and tractability of each target.

Phylogenomic analyses across *Plasmodium* spp. and comparative genomic studies in humans have led to the identification of new drug targets in *P. falciparum*. Identification of essential genes (targets) responsive to specific inhibitors led to the discovery of 40 potential drug targets, which includes known ones such as calcium dependent protein kinase and previously unknown ones such as phosphoisomerase and carboxylase (Ludin et al., 2012). Comparing the transcriptomes of six *Plasmodium* spp. during blood stage infection revealed about 800 genes that have similar expression patterns across species, among which 240 were demonstrated to be druggable by online drug target prioritization databases (Hoo et al., 2016). Similarly, genomic and transcriptomic analyses have been carried out with other pathogens with encouraging results in fungi (Kaltdorf et al., 2016), bacteria (Turab Naqvi et al., 2017), and viruses (Dapat and Oshitani, 2016).

In vaccine target identification, pathogen genomes are being scanned in a bid to identify genes encoding proteins or molecules with vaccine candidate properties such as low antigenic variation, polymorphism, and immunogenicity (Masignani et al., 2002; De Groot et al., 2008). Despite the success of whole-organism vaccines such as those for polio, whole-organism vaccines for pathogens such as *Plasmodium spp.*, *Mycobacterium spp.* and HIV remain a challenge (Doolan et al., 2014; Proietti and Doolan, 2015). Genomics offers a potential way around this challenge through the discovery of immunogenic antigens using whole-genome scans (Doolan et al., 2014; Proietti and Doolan, 2015). Here, omics techniques and bioinformatics tools are used to determine genes or proteins that are involved in the virulence of the pathogen and pathogenesis of the disease by comparing, for example, attenuated and pathogenic disease agents. Algorithms can be used to predict T cell epitopes or regions with high affinity within HLA molecules in translated peptides found in databases (Grubaugh et al., 2013; Davies et al., 2015) in order to inform the choice of the right antigens for vaccine design. Omics technologies have been reviewed in the context of vaccine target identification by He (2012).

Most of the tools used for epitope identification rely on statistics and machine learning. Some of them include servers to predict MHC-binding, peptides namely RANKPEP (Reche et al., 2004), which uses Position Specific Scoring Matrices (PSSMs), and nHLAPred^6^ (Bhasin and Raghava, 2007), based on Artificial Neural Networks (ANNs) and quantitative matrices among others. Some severs are specific for B-cell epitope prediction, such as Bcepred^7^ (Saha and Raghava, 2004), ABCpred^8^ (Saha and Raghava, 2006), and BepiPred^9^ (Jespersen et al., 2017). These tools work based on the physicochemical properties and location of the peptides. They function alongside epitope-containing databases such as Swiss-Prot, SYFPEITHI, and IEDB (Fleri et al., 2017). The list of tools, methods and databases mentioned here is not exhaustive, however, they have been extensively reviewed elsewhere (Soria-Guerra et al., 2015).

Nowadays, due to advances in the fields of computer sciences, genomics, proteomics, bioinformatics and management of patients’ health records, etc., there seems to be a paradigm shift from generalized medicine to personalized therapy (Sorber et al., 2017). For example, many drugs are metabolized by cytochrome P450 enzymes with drug action depending on the expressed gene variant (BlueCross and BlueShield Association, 2004; Daly et al., 2006). Moreover, malaria patients with glucose-6-phosphate (G6p) deficiency have been reported with severe complications such as cardiotoxicity and acute hemolytic anemia following treatment with quinidine gluconate (Damhoff et al., 2014). These complications have been described as a consequence of inherited (X-linked trait) mutations in the *g6p* gene (Luzzatto and Seneca, 2014). These mutations do not cause the complete loss of the G6P enzyme but instead affect its stability and level in red blood cells (Luzatto et al., 2001). In the same line rifampicin, which is the drug of choice for TB treatment, is transported after administration by a human anion transporter encoded by the *SLCO1B1* gene. Studies have shown that mutations in the *SLCO1B1* gene, namely rs11045819 and rs4149032, are associated with decreased RIF plasma levels in South-African populations (Weiner et al., 2010; Chigutsa et al., 2011; Gengiah et al., 2014). However, this finding could not be replicated in Malawian and South Indian populations, implying that this could be population-specific (Ramesh et al., 2016; Sloan et al., 2017). These show, in a nutshell, the implication of genomics and bioinformatics in drug discovery and precision therapy (Hamburg and Collins, 2010; Rabbani et al., 2016).

## Challenges and Opportunities in Conducting Omics and Bioinformatics Studies in Africa

Bioinformatics is increasingly becoming an important cornerstone in contemporary research on infectious diseases (Mulder et al., 2017), where Africa has the highest morbidity and mortality but less genomics research output compared to other regions of the world (Fatumo et al., 2014; Karikari, 2015). This slow pace of genomics research output is due to several challenges in omics and bioinformatics research facilities in Africa; three of the major ones are briefly discussed.

### Inadequate Infrastructure

Bioinformatics and genomics analysis require powerful computers and a reliable source of electricity for large data storage and high throughput analyses (H3Africa Consortium et al., 2014). With the exception of some South African universities, most sub-Saharan African universities lack high performance computing facilities (Karikari et al., 2015; Mulder et al., 2016). There is also a limitation of high-speed internet for sharing data and accessing bioinformatics databases and repositories (Fatumo et al., 2014; Karikari, 2015). This hinders the application of cloud-based web services which could have circumvented the need for local high-performance computing facilities (Navale and Bourne, 2018). Furthermore, few research institutions in Africa have sequencing facilities and therefore resort to sequencing abroad through collaborations. Such collaborations often result in a loss of ownership of the data and resulting publications usually have the external collaborators as lead and correspondence authors. Notable efforts being made to bridge this infrastructural gap include the installation of high-performance computers (HPCs) at The Developing Excellence in Leadership and Genetics Training for Malaria Elimination in sub-Saharan Africa (DELGEME) at the University of Science Technique and Technologies of Bamako, Mali, the West African Centre for Cell Biology of Infectious Pathogens (WACCBIP), University of Ghana and the Medical Research Council Unit, The Gambia at the London School of Hygiene and Tropical Medicine, to support storage and high throughput analyses of genomic data. These HPC facilities are complemented by NGS sequencing facilities at WACCBIP and MRC in addition to some institutions in East Africa such the International Livestock Research Institute (ILRI-Kenya). This infrastructural development, and pressure from initiatives such as Human Heredity and Health in Africa (H3Africa), will hopefully serve as a springboard for Africa to increase her involvement in the study design, sample collection, analysis and ownership of data rather than just collecting samples for international collaborators.

### Lack of Training Opportunities and Well-Structured Bioinformatics Courses

Until the recent introduction of bioinformatics training courses by H3ABioNet, there were limited bioinformatics training courses in Africa. Such training programs were mostly short courses organized by local bioinformaticians with support from experts in the field across Africa and other external collaborators (Gurwitz et al., 2017). Very few African universities have structured bioinformatics courses, most of these universities are South African, while some are North African and few are in sub-Saharan Africa (Bishop et al., 2015). The DELGEME, through funding from the Wellcome Trust, is also providing funding for Master of Science courses in bioinformatics, which are mostly done in South Africa. The other form of bioinformatics training is through local capacity building, which institutions organize for staff with support usually through North-South collaborations and transfer of expertise. However, the downside of short courses is that there is no mentorship beyond the course, which hinders consolidation of the knowledge gained. In addition to these, some organizations working predominantly on crop production, such as the International Institute of Tropical Agriculture Bioscience Center^10^ and Consultative Group on International Agricultural Research institute^11^, offer short bioinformatics training opportunities to African scholars. Sometimes some students from Africa get training from European universities, but the challenge is that most of the trainees do not come back to join local institutions because of poor infrastructures. Furthermore, there is a disconnect between biologists and other scientific disciplines such as computer science, statistics and mathematics in most African universities. This affects multidisciplinary research, which is crucial in modern-day infectious disease research. Ultimately, the lack of well-structured bioinformatics curricula hampers the development and maintenance of highly needed experts in the field in Africa, since they often move to Europe and North America for better career prospects.

### Limited Research Funding

A major challenge to research on the African continent is the lack of funding for biomedical research. Current research is mainly funded from international donors, with limited or no funding from national governments and African regional bodies such as the African Union (Hamburg and Collins, 2010; Karikari, 2015). However, a few countries such as South Africa, through the South Africa’s National Research Foundation and Medical Research Council, do provide funding for genomics research projects (Karikari et al., 2015). Until the initiation of H3Africa, through funding from the National Institute of Health (United States) and the Wellcome Trust (United Kingdom), there was limited to no funding for genomics and bioinformatics in Africa (Adoga et al., 2014; Mulder et al., 2017).

## Conclusion and Perspective

Herein we highlight how genomics and bioinformatics has contributed to our understanding of infectious diseases of significant health concern, ranging from bacterial and viral to parasitic infections, as well as their applications to drug and vaccine target identification. This ranges from understanding pathogenesis, host systemic responses and host-pathogen interactions to identification of prognostic and diagnostic markers. However, in Africa, despite the high morbidity and mortality due to infectious diseases, there is limited expertise in the field of bioinformatics and hence limited bioinformatics research output in terms of publications. Thus, there is a need to strengthen training and capacity building in bioinformatics in Africa to improve infectious disease genomics and host-pathogen genomics on the continent. This can be achieved through the establishment of well-structured courses, mentorship for junior and trainee bioinformaticians and better career prospects to maintain trained bioinformaticians on the continent.

## Author Contributions

All authors listed contributed substantially to the intellectual, writing and editing, and approved the manuscript for publication.

## Conflict of Interest Statement

The authors declare that the research was conducted in the absence of any commercial or financial relationships that could be construed as a potential conflict of interest.

## Footnotes

1 https://www.malariagen.net/

2 http://geneontology.org/

3 http://www.ncbi.nlm.nih.gov

4 http://EuPathDB.org

5 http://www.wormbase.org

6 http://www.imtech.res.in/raghava/nhlapred/

7 http://www.imtech.res.in/raghava/bcepred/

8 http://www.imtech.res.in/raghava/abcpred/

9 http://www.cbs.dtu.dk/services/BepiPred/

10 http://bioscience.iita.org/index.php/en/services/bioinformatics

11 https://www.cgiar.org/
